# Hospital Meetings, &c.

**Published:** 1899-03-18

**Authors:** 


### HOSPITAL MEETINGS, &C.

### WEST HAM HOSPITAL.

The annual general meeting of the governors and subscribers to the West Ham Hospital was held in the dispensing room of the institution, West Ham Lane, Stratford, E., on Wednesday (8th inst.), Alderman Hay presiding. The Secretary (Mr. Leonard D Rea), in dealing with the report of the committee of management, pointed out that the growing usefulness of the hospital might be best judged by the constant increase [illegible] the number of in-patients, who in 1895 were 441, whilst for the past year they had numbered 744. Of the cases detained during 1898 621 were surgical and 123 medical; 439 were cured, 213 relieved, 44 died, and 48 remained in hospital at the close of the year. Surgical operations performed numbered 228, and the committee pointed out in their report that the absolute necessity for an accident hospital in that "great community of toil" could not now be questioned. The out-patients' department continues to treat over 20,000 cases in the year. The average cost of each in-patient was £4 13s., and of each out-patient 1s. 1d., the average number of days an in-patient remained was 19, the daily average of patients throughout the year being 39. Mr. Jonathan Hutchinson, jun., F.R.C.S., has accepted the position of honorary consulting surgeon, rendered vacant by the resignation of Mr. Frederick Treves, Mr. P. J. S. Nicoll succeeding to the position of honorary surgeon rendered vacant by the resignation of Mr. C. Sanders. Mr. J. Oliphant Goldie has retired on completion of his term of office as senior house surgeon, and the committee have appointed Mr. F. W. Rowland and Mr. R. Niven to the senior and junior post respectively. The accounts for the year showed that the total ordinary income was £4,271 1s. 9d., and the total ordinary expenditure £4,681 12s. (the extraordinary expenditure amounting to only £15 1s. 8d. additional), the deficit being £263 8s. 1d. The committee emphasise the fact that this is the first time in the history of the hospital that a year has closed in debt, the explanation being that whilst the growth of the population of the district has brought an increased number of patients, the expenses necessary to efficiency have increased; at the same time the income has not improved. The committee in conclusion strongly appeal for help. The report having been put to the meeting by the chairman it was adopted.

### HAMPSTEAD HOSPITAL.

The seventeenth annual meeting of governors and subscribers of the Hampstead Hospital was held at the Vestry Hall, Haverstock Hill, on Saturday last, 11th inst., Mr. J. McMillan presiding in the absence of Mr. Malcolm, the treasurer, who, it was announced with much regret, was suffering from influenza. From the report of the council it appeared that the income for the year was £3,546 (including £170 in hand), and the expenditure £3,519, leaving a credit balance of £26. Of these sums the ordinary income amounted to £3,330, and the ordinary expenditure to £3,213; added to which there was a legacy of £45 on the one side and £306 for new buildings and repairs on the other—part of this latter being provided for by a special donation. There was an increase of £250 in the annual subscriptions. During the year 327 patients were admitted as against 305 in 1897, besides 261 minor casualties not requiring to stay in the hospital more than one day. In the out-patients' department there were 2,327 attendances. The festival dinner in April at the Grand Hotel was marked by the definite starting of a scheme for building a new hospital. In furtherance of this great efforts are being made, and the council announce that already £3,000 has been promised. The total sum required is about £20,000. In presenting the report the Chairman said they now saw their way to placing the hospital in the position it ought to occupy. Their success mainly depended on eliciting the support of the ladies of Hampstead, and the Ladies' Association had done what no other organisation could have done. Without them their position might have been quite a different one, they might even have been shut up; now, however, they had every reason to hope that before very long they would find themselves in more commodious and suitable quarters, with a building worthy of their locality and surroundings. In June they were to have a garden party at Golder's Hill, at which, if her engagements would allow, Her Royal Highnes Princess Christian would come forward again to help them

The Chairman concluded his remarks with a warm tribute to the medical staff and the nurses, and also to the untiring energy and ability of his friend and colleague Mr. Owthwaite, the hon. secretary. Dr. Heath Strange read the medical report, and Mr. Blythe that of the Ladies' Association, and after the re-election of the council for the ensuing year, with power to add to their number, had been adopted, and Sir Richard Temple and Mr. Edward Bond, M.P., elected trustees, a vote of thanks to the chairman closed the proceedings.

### FRENCH HOSPITAL AND DISPENSARY.

On Thursday, 9th inst., Dr. A. Vintras presided at the thirty-first annual general meeting of the French Hospital and Dispensary, Shaftesbury Avenue. The report and accounts for the past year were adopted without discussion. From them it appeared that the ordinary income of the hospital was £3,896 and the ordinary expenditure £3,650. Extraordinary income (legacies, &c.) amounted to £3,209, and extraordinary expenditure to £36, chiefly on account of the electric light. The number of in-patients treated during the year was 764, as compared with 711 in 1897. The out-patients numbered 4,833, as against 4,722 in the previous year. With the exception of the opening of a convalescent home at Brighton in October by the then French Ambassador, the Baron de Courcel, the French Hospital is one of those happy institutions which have no history beyond another year's record of good work satisfactorily done—a fact which accounts for the limited attendance at the meeting. As the Chairman remarked, "So long as they are satisfied all is right, our friends do not trouble to come; if anything were going wrong, they would be here."

### ST. PETER'S HOSPITAL.

In the absence of Mr. Bevan, the treasurer, through indisposition, Mr. Thomas Christy presided at the thirty-first annual meeting of St. Peter's Hospital for Stone and Urinary Diseases, Henrietta Street, on Thursday, 9th inst.

From the report presented by the Committee of Management it appeared that during 1898 502 in-patients were admitted, the largest number ever treated in one year, and 28 more than in 1897, when the previous record was established. Many patients had to be kept waiting till a vacancy occurred owing to the small number of beds. Of the 502 patients treated, 483 were cured or relieved, and 19 died; some of the latter, having been operated on elsewhere, were in an enfeebled condition when admitted. The out-patients numbered 4,773, rather above the average; the attendances registered were 36,631. No less than 621 medical practitioners and students attended the practice of the hospital during the year, showing that the work is valued by those engaged in the study of this special branch of surgery. The committee expressed regret and disappointment at the discontinuance of the usual grant from the Hospital Saturday Fund owing to new rules having been adopted excluding from participation hospitals whose patients contribute 75 per cent. towards the cost of their maintenance. In June Sir Trevor Lawrence and Mr. N. C. Macnamara visited the hospital as a deputation from the Prince of Wales's Fund. On leaving they expressed themselves as satisfied with all the arrangements, and the grant was increased from £26 5s. to £50. During the summer the council of the Hospital Sunday Fund through their vice-president, Sir Sydney Waterlow, also inspected the hospital, with a similar result of an increased award. The ordinary income amounted to £3,513 and the ordinary expenditure to £3,430. The extraordinary expenses were £104, so that the year's accounts show an excess of expenditure over receipts of £20. Satisfaction was expressed that the falling off in subscriptions and donations which occurred during the Jubilee year had not continued, the Chairman remarking that they had practically regained their old position. He complained bitterly of what he described as the contemptible character of the grant of the Prince of Wales's Fund to a hospital such as theirs, receiving, as it did, so large a proportion of patients requiring operations of the most important character. This ought to be recollected when heads were being counted with a view to the allocation of grants. The continued increase in the number of students attending showed the estimation in which the work of the hospital was held among those best able to judge of its value. He appealed to business men to help them by joining the Committee of Management.

In seconding the adoption of the report Mr. Edmonds emphasised the statement that mere counting of heads was no fair gauge of the hospital's work. In general hospitals quite half and often seven out of twelve cases were medical; with them all were surgical, and in 84 per cent. operations were performed, so that their work was peculiar and necessarily more expensive, and there could be no comparison if figures only were taken into account. As a constant visitor himself he could say he did not believe any institution was managed with greater care or economy.

Mr. Hurry Fenwick, F.R.C.S., moved the adoption of the medical report, which he characterised as satisfactory, in spite of the fact that 19 deaths had occurred. He knew that those who looked at it from the professional point of view would admit that their success had been great.

Mr. Reginald Harrison, F.R.C.S., seconded, and bore testimony to the great credit due to the matron for her care in the training and selection of their nurses, upon whom so much depended.

In moving a vote of thanks to the medical staff Mr. Edmonds said the money value alone of the operations performed by them would amount to many hundreds of thousands of pounds. Their work was not only done with very great skill, but with very great kindness; and the gratitude of the whole profession was due to them for the way in which every facility was given for others to learn all that their experience was teaching them.

The resolutions were carried *nem. con.*, and a hearty vote of thanks to the chairman, not only for presiding on that occasion, but for his 40 years of tireless devotion to the interests of the institution, closed the proceedings.

### IMPROVEMENTS AT THE NEW HOSPITAL FOR WOMEN.

The new ward, named the "Grace Chadburn," occupies the old nurses' quarters at the top of the hospital. It is separated from the main building by a cross ventilated passage. A similar passage isolates the bath-room and lavatories at the farther side. It has a sloping ceiling irregularly broken by numerous windows and ventilators, in addition to the goodly number in the walls proper. The floor is polished teak, the cement walls are tinted buff and cream, and the paint is of a soft sage-green colour. There are easy chairs, a couple of oak tables, &c. It is warmed by two small open stoves standing well forward with thick flues, of which a considerable length is inside the ward. There is a small, convenient ward kitchen and an examining-room.

The operating room occupies a central position in the hospital. Nearly the whole of one side is glass, whilst a great portion of the ceiling is glass also. The walls are of white cement, the floor white mosaic, the corners are rounded, and the furnishings modern. The new operation table is the gift of Mrs. Scharlieb. The shelves are glass on metal brackets, and so are the dressing tables. The lavatory basin is supplied with hot and cold water, which passes through a mixing pipe to secure command of the temperature of the water. The room is heated by an open fire with a ventilating flue above, and by radiating coils. The sterilising apparatus, also Mrs. Scharlieb's gift, is placed in a hooded recess, well fitted with ventilators to carry off the steam and heat. There is no students' gallery.

